# Species diversity of fishes in the Dingqu River Basin, tributary of the upper Yangtze River, China

**DOI:** 10.3897/BDJ.9.e76669

**Published:** 2021-11-22

**Authors:** Yingchun Xing, Jie Bai, Huiqin Li, Baoxiang Liu, Yahui Zhao

**Affiliations:** 1 Resource and Environmental Research Center, Chinese Academy of Fishery Sciences, Beijing, China Resource and Environmental Research Center, Chinese Academy of Fishery Sciences Beijing China; 2 Key Laboratory of the Zoological Systematics and Evolution, Institute of Zoology, Chinese Academy of Sciences, Beijing, China Key Laboratory of the Zoological Systematics and Evolution, Institute of Zoology, Chinese Academy of Sciences Beijing China

**Keywords:** Qinghai-Tibet Plateau, hydropower, distribution, habitat condition, endemism, endangered species

## Abstract

**Background:**

The Dingqu River Basin, a major tributary of the upper Yangtze River, is located at southeast edge of the Qinghai-Tibet Plateau of China. The fishes of this plateau constitute a major faunal component of this basin, particularly Schizothoracinae (Cypriniformes: Cyprinidae) and *Triplophysa* (Cypriniformes: Nemacheilidae). Hydropower development is an impact that affects natural habitats and biological resources of the upper Yangtze River and this has led to a decrease in biodiversity. This study investigated the species diversity of fishes of the Dingqu River Basin and accumulated basic data for conservation of biodiversity and assessment of ecological health of the upper Yangtze River.

**New information:**

The upper streams of the Jinshajiang River harbours numerous endemic fish species in China. Most of them belong to the Qianghai-Tibet Plateau fish fauna. However, while the fish species of the Jinshajiang River have been well studied, there is still a gap in the research on fish species diversity of the Dingqu River Basin tributary. This study provides information for 18 native fish species belonging to two orders, four families, three subfamilies and eight genera, and is the first complete record of fishes in the Dingqu River Basin, a primary tributary of the Yangtze River. Supplemental information of species diversity in the Jinshajiang River is also provided. The study includes two datasets, which present taxonomic, distribution, habitat condition, endemism and threat information for each species collected from the Dingqu River Basin and historical literature, respectively. In particular, these fish species all have limited distribution within the south-eastern Qinghai-Tibet Plateau areas of China and could determine the importance of habitat protection for the upper Yangtze River.

## Introduction

The Jinshajiang River (https://www.feow.org/ecoregions/details/764) is the upper section of the upper Yangtze River, flowing through Qinghai, Tibet, Yunnan, Guizhou and Sichuan Provinces of the south-eastern plateau of China with a length of 2308 km, area of 3.5×10^5^ km^2^ and elevational difference of 3300 m (Zhu 1981, Wu and Wu 1992). Fish is a significant biological component of the river and research on its species diversity remains a high priority. Most early records identified 30 fish species distributed in the Jinshajiang River and seven species in the upper section (Cao et al. 1981). Multiple studies (Chen and Chu 1989, Chu and Chen 1990, Wu and Wu 1990, Ding 1994) have resulted in the recent recognition of 198 fish species in the Jinshajiang River (Zhang et al. 2019). The Dingqu River Basin lies in southeast edge of the Qinghai-Tibet Plateau, is the major tributary on the left bank of the upper Yangtze River, and is the largest tributary of the Jinshajiang River. The length of the main stream of the Dingqu River Basin is 226 km with an area of 12213 km^2^ (Xu 2020). Although this tributary occupies a very important geographic location, the fish species diversity and distribution information have not as yet been systematically researched.

Undoubtedly, as the largest river both in China and Asia and globally the third largest river, the Yangtze River plays a doubly important role in providing hydropower for human society development and habitats for aquatic organisms (Floehr et al. 2013, Zeng et al. 2013). Its fish fauna is one of the richest worldwide at 426 species, amongst which 175 species are endemic to the river (Fu et al. 2003, Xing et al. 2016). Hydropower projects constructed on the upper stream have brought a serious threat to aquatic biodiversity of the Yangtze River; therefore, assessment of the biodiversity has become especially important and could provide basic data for scientific understanding and utilisation of natural resources. For a long time, there has been no focused survey on fish species diversity of the Dingqu River Basin, and therefore lacking important data. Results of our investigation will contribute to overall fish data in the upper Yangtze, and especially for the upper Jinshajiang River.

## Sampling methods

### Sampling description

The Dingqu River flows through four counties (Litang, Batang, Xiangcheng and Derong) from north to south, flows into the Mayihe River in Derong County and then converges with the Shuoqu River into the Jinshajiang River, which forms the Dingqu River Basin (Xu 2020). Fish specimens were collected at one locality of the Dingqu River in May 2012, four localities (two in the Dingqu River, one in the Mayihe River and one in the Shuoqu River) in September 2012, 13 localities (four in the Dingqu River, two in the Mayihe River and seven in the Shuoqu River) in September 2019, and 15 localities (seven in the Dingqu River and eight in the Shuoqu River) in June and July 2020, respectively. Fish specimens were collected by gillnets, cast nets and traps at each habitat (swift-flowing waters, riffles, running waters and pools). Meanwhile, longitude, latitude and elevation of each sampling locality and fundamental water parameters (temperature, pH, dissolved oxygen and conductivity) were recorded by use of a hand-held multi-parameter water quality analyser (YSI Pro Plus), as well as photos of habitats being taken. The specimens were preserved in 95% ethanol in the field and then deposited in 75% ethanol in the National Zoological Museum, Institute of Zoology, Chinese Academy of Sciences (ASIZB).

### Step description

Fish species were identified by at least two professional fish taxonomists and referenced against literature on fish species of the Jinshajiang River, the Tibet Plateau and Hengduan Mountain (Wu and Wu 1992, Ding 1994, Chen 1998, Zhang et al. 2019). Valid species names were in accordance with the taxonomic literature (Zhang et al. 2020).

## Geographic coverage

### Description

**Figure 1.**
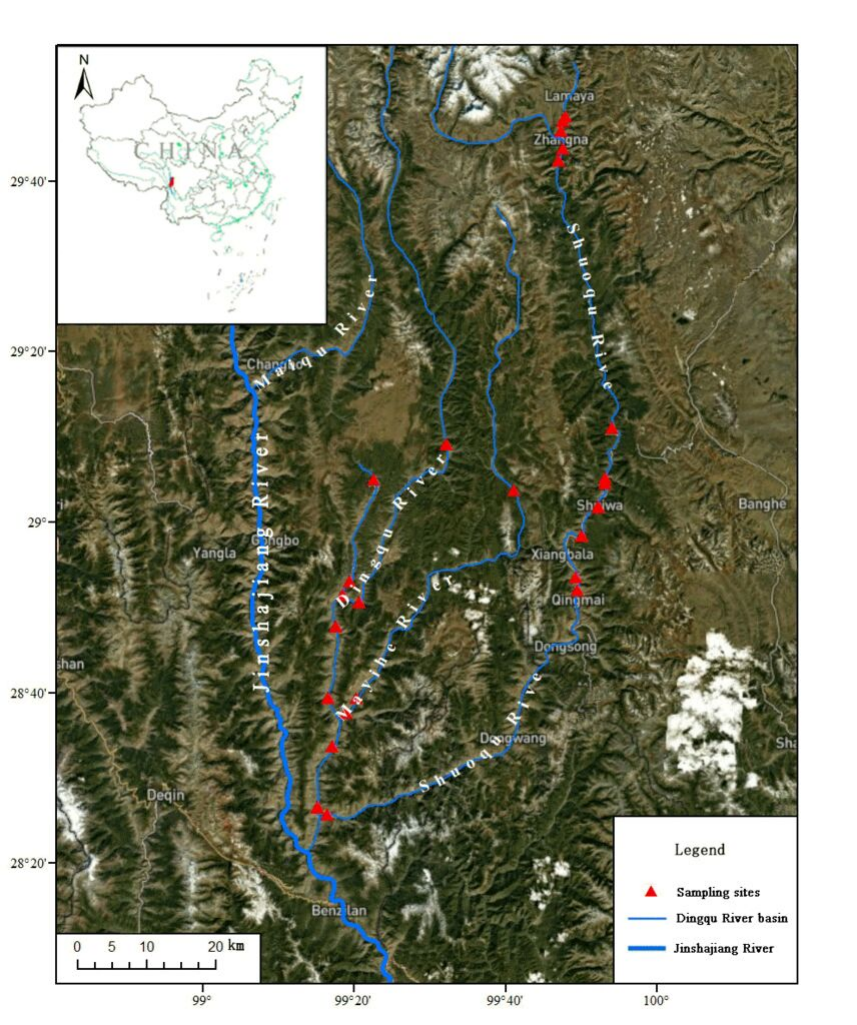
Location of the sampling sites.

**Figure 2.**
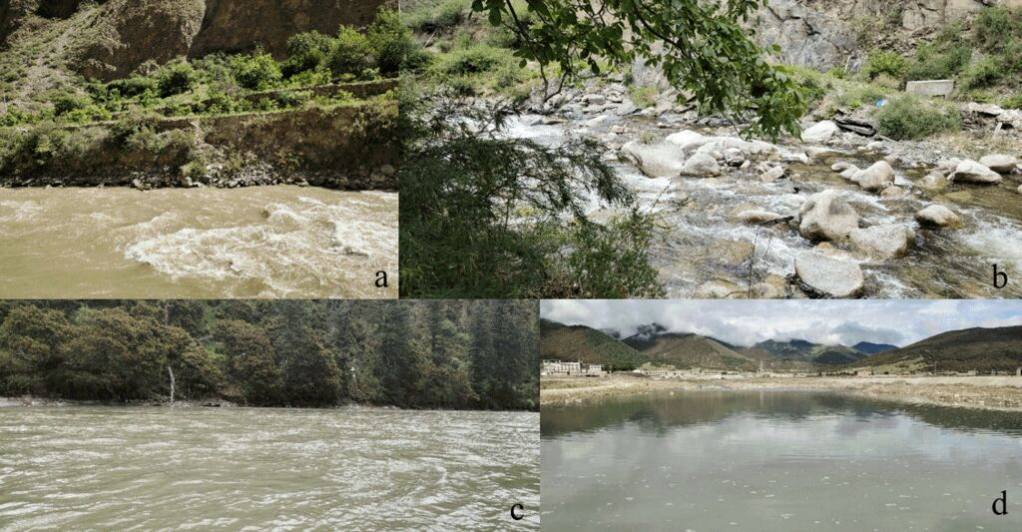
Photos of habitats of the Dingqu River basin. **a** swift-flowing waters; **b** riffles; **c** running waters; **d** pools.

We surveyed all three main streams of the Dingqu River Basin, i.e. the Dingqu, Mayihe and Shuoqu Rivers (Fig. 1), covering habitats such as swift-flowing waters, riffles, running waters and pools (Fig. 2). The investigated water area is 26.74 hectares calculated using ArcGIS 10.1 software. Historical records of fishes in the Dingqu River Basin were also collected.

### Coordinates

28.428 and 29.791 Latitude; 99.910 and 99.253 Longitude.

## Taxonomic coverage

### Description

**Figure 3.**
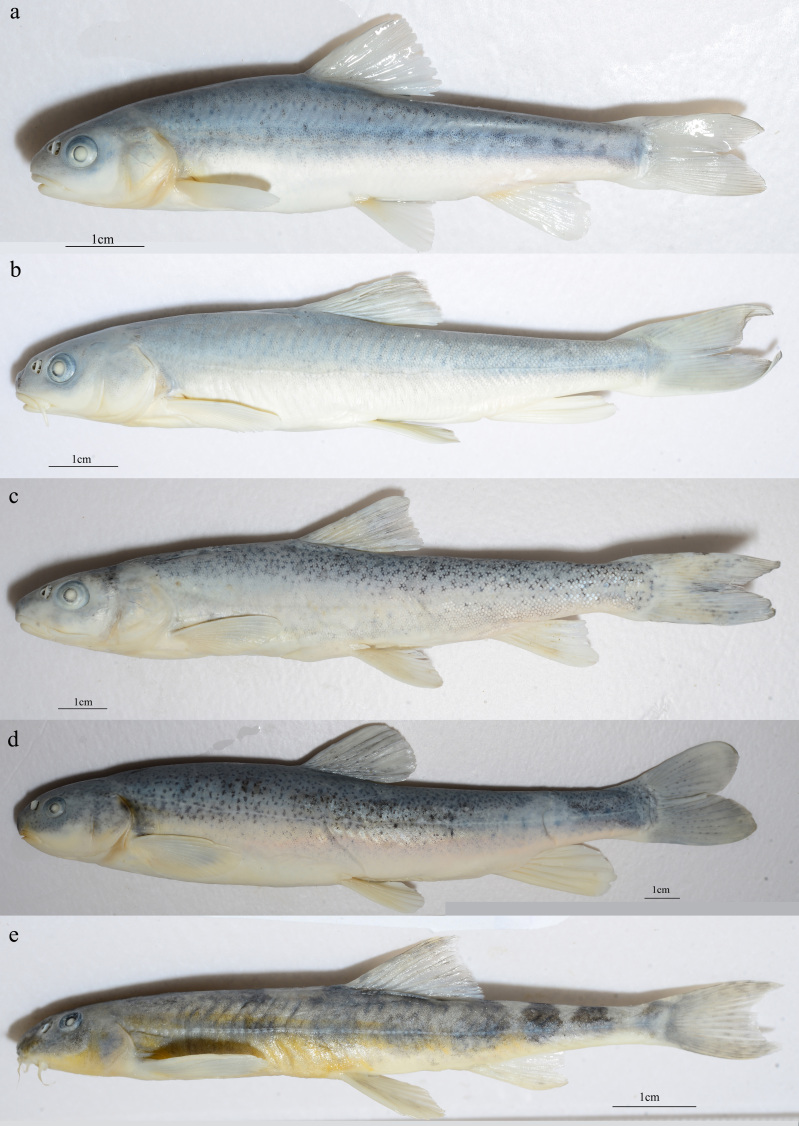
Specimen photos of some fish species collected from the Dingqu River Basin. a. *Gymnocyprispotaninifirmispinatus* Wu & Wu, 1988; b. *Ptychobarbuschungtienensisgezaensis* (Huang & Chen, 1986); c. *Ptychobarbuskaznakovi* Nikolsky, 1903; d. *Schizopygopsismalacanthusmalacanthus* Herzenstein, 1891; e. *Triplophysapseudostenura* He, Zhang & Song, 2013.

In total, two orders, four families, eight genera and 18 native fish species were collected in our study on the Dingqu River Basin. Specimen photos of representative fish species are presented in Fig. 3.

### Taxa included

**Table:** 

Rank	Scientific Name	
kingdom	Animalia	
phylum	Chordata	
class	Actinopterygii	
order	Cypriniformes	
order	Siluriformes	
family	Cyprinidae	
family	Nemacheilidae	
family	Sisoridae	
family	Botiidae	
subfamily	Schizothoracinae	
subfamily	Nemacheilinae	
subfamily	Botiinae	
genus	*Gymnocypris*	
genus	*Schizopygopsis*	
genus	*Schizothorax*	
genus	*Ptychobarbus*	
genus	*Gymnodiptychus*	
genus	*Triplophysa*	
genus	*Paramisgurnus*	
genus	*Euchiloglanis*	
species	*Gymnocyprispotaninifirmispinatus* Wu & Wu, 1988	
species	*Paramisgurnusdabryanus* Dabry de Thiersant, 1872	
species	*Ptychobarbuschungtienensisgezaensis* (Huang & Chen, 1986)	
species	*Ptychobarbuskaznakovi* Nikolsky, 1903	
species	*Schizopygopsismalacanthusmalacanthus* Herzenstein, 1891	
species	*Schizothoraxchongi* (Fang, 1936)	
species	*Schizothoraxgrahami* (Regan, 1904)	
species	*Schizothoraxwangchiachii* (Fang, 1936)	
species	*Triplophysableekeri* (Sauvage & Dabry de Thiersant, 1874)	
species	*Triplophysabrevicauda* (Herzenstein, 1888)	
species	*Triplophysadaqiaoensis* Ding, 1993	
species	*Triplophysaleptosoma* (Herzenstein, 1888)	
species	*Triplophysapseudostenura* He, Zhang & Song, 2013	
species	*Schizothorax kozlovi Nikolsky*, 1903	
species	*Schizothoraxdolichonema* Herzenstein, 1889	
species	*Gymnodiptychuspachycheilus* Herzenstein, 1892	
species	*Euchiloglanisdavidi* (Sauvage, 1874)	
species	*Euchiloglaniskishinouyei* Kimura, 1934	

## Temporal coverage

**Single date:** 2012-5-04; 2012-9-15; 2019-9-04; 2020-6-26.

**Data range:** 2012-9-12 – 2012-9-13; 2020-9-08 – 2020-9-11; 2020-6-18 – 2020-6-20; 2020-7-02 – 2020-7-06.

## Usage licence

### Usage licence

Creative Commons Public Domain Waiver (CC-Zero)

## Data resources

### Data package title

Fish taxon-occurrences of Dingqu River, Jinshajiang River, China

### Number of data sets

2

### Data set 1.

#### Data set name

Collected fish taxon-occurrences of Dingqu River

#### Data format

Darwin Core

#### Number of columns

29

#### Description

Suppl. material 1

**Data set 1.** 

Column label	Column description
occurrenceID	An identifier for the Occurrence.
catalogNumber	An identifier for preserved specimens.
basisOfRecord	The specific nature of the data record.
eventDate	The date during which an Event occurred.
scientificName	The full scientific name.
kingdom	The full scientific name of the kingdom in which the taxon is classified.
phylum	The full scientific name of the phylum in which the taxon is classified.
class	The full scientific name of the class in which the taxon is classified.
order	The full scientific name of the order in which the taxon is classified.
family	The full scientific name of the family in which the taxon is classified.
subfamily	The full scientific name of the subfamily in which the taxon is classified. No subfamily is represented by NA.
genus	The full scientific name of the genus in which the taxon is classified.
taxonRank	The taxonomic rank of the most specific name in the scientificName as it appears in the original record.
ownerInstitutionCode	The name (or acronym) in use by the institution having ownership of the object(s) or information referred to in the record.
individualCount	The number of individuals represented present at the time and location of the Occurrence.
recordedBy	A list (concatenated and separated) of names of people, groups or organisations who record the information of the Taxon when collected.
identifiedBy	A list (concatenated and separated) of names of people, groups or organisations who assigned the Taxon to the subject.
decimalLatitude	The geographic latitude (in decimal degrees, using the spatial reference system given in geodeticDatum) of the geographic centre of a Location.
decimalLongitude	The geographic longitude (in decimal degrees, using the spatial reference system given in geodeticDatum) of the geographic centre of a Location.
maximumElevationInMetres	The geographic elevation (in metres, using the spatial reference system given in geodeticDatum) of the geographic centre of a Location.
geodeticDatum	The geographic information system (GIS) upon which the geographic coordinates given in decimalLatitude, decimalLongitude and meterElevation are based.
coordinateUncertaintyInMetres	The horizontal distance (in metres) from the given decimalLatitude and decimalLongitude describing the smallest circle containing the whole of the Location. Leave the value empty if the uncertainty is unknown, cannot be estimated or is not applicable (because there are no coordinates). Zero is not a valid value for this term.
locality	The specific description of the county from where specimens are collected.
country	The name of the country or major administrative unit in which the Location occurs.
stateProvince	The name of the next smallest administrative region than country (state, province, canton, department, region etc.) in which the Location occurs.
municipality	The full, unabbreviated name of the next smallest administrative region than county (city, municipality etc.) in which the Location occurs.
waterBody	The name of the water body in which the Location occurs.
habitat	A category or description of the habitat in which the Event occurred.
dynamicProperties	A list of descriptions of endemism of taxon in the Jianshajiang River or the Yangtze River ("yes" refers to "endemic", "no" refers to "non-endemic") and description of degree of threat of taxon according to China’s Red List of Biodiversity: Vertebrates (Zhang and Cao 2021). Degree of threat is recommended to be ranked as Critically Endangered (CR), Endangered (EN), Vulnerable (VU), Near Threatened (NT), Least Concern (LC), Data Deficient (DD) and Not Evaluated (NE).

### Data set 2.

#### Data set name

Historical fish taxon-occurrences of Dingqu River

#### Data format

Darwin Core

#### Number of columns

18

#### Description

Suppl. material 2

**Data set 2.** 

Column label	Column description
occurrenceID	An identifier for the Occurrence.
scientificName	The full scientific name.
kingdom	The full scientific name of the kingdom in which the taxon is classified.
phylum	The full scientific name of the phylum in which the taxon is classified.
class	The full scientific name of the class in which the taxon is classified.
order	The full scientific name of the order in which the taxon is classified.
family	The full scientific name of the family in which the taxon is classified.
subfamily	The full scientific name of the subfamily in which the taxon is classified. No subfamily is represented by NA.
genus	The full scientific name of the genus in which the taxon is classified.
taxonRank	The taxonomic rank of the most specific name in the scientificName as it appears in the original record.
country	The name of the country or major administrative unit in which the Location occurs.
stateProvince	The name of the next smallest administrative region than country (state, province, canton, department, region etc.) in which the Location occurs.
municipality	The full, unabbreviated name of the next smallest administrative region than county (city, municipality etc.) in which the Location occurs.
locality	The specific description of the place.
habitat	A category or description of the habitat in which the Event occurred.
dynamicProperties	A list of descriptions of endemism of taxa in the Jianshajiang River or the Yangtze River ("yes" refers to "endemic", "no" refers to "non-endemic") and description of degree of threat of taxon according to China’s Red List of Biodiversity: Vertebrates (Zhang and Cao 2021). Degree of threat is recommended to be ranked as Critically Endangered (CR), Endangered (EN), Vulnerable (VU), Near Threatened (NT), Least Concern (LC), Data Deficient (DD) and Not Evaluated (NE).
associatedReferences	The origins of historical data come from recommended best reference to academic paper, book or official database.
language	The language used by dataset.

## Additional information

Fish composition of the Dingqu River Basin presents typical Qinghai-Tibet Plateau fauna, with Schizothoracinae (Cypriniformes: Cyprinidae) and *Triplophysa* (Cypriniformes: Nemacheilidae) being mainly distributed in the Qinghai-Tibet (Wu and Wu 1992). Amongst the total of 18 native fish species, all species are endemic to China; 16 species (89% of total) are endemic to the upper Yangtze River and eight species (44% of total) are endemic to the Jinshajiang River. Based on Red List of China’s Vertebrates (Zhang and Cao 2021), four species are “Endangered (EN)”, five species are “Vulnerable (VU)”, four species are “Least Concern (LC)”, three species are “Data Deficient (DD)” and two species are “Not Evaluated (NE)”. In addition, the distribution of fish indicates corresponds with habitat conditions. For example, the genus *Paramisgurnus* has limited distribution at riffles, the genus *Euchiloglanis* is generally found living at riffles with rapid water movement, the genus *Triplophysa* is found at the bottom of rapid waters and the genus *Schizothorax* lives in running waters.

The subfamily Schizothoracinae of the Dingqu Basin consists of higher species richness (10 species, 56% of total species) and their morphological diversity shows all specialisation levels of this subfamily. Three specialisation levels of morphology for Schizothoracinae were proposed: a. Original group, where the whole body is covered by scales or with partial degeneration; b. Specialised group, whose body scales are partially or all degenerated; and c. Highly specialised group, whose body scales all degenerated (Cao et al. 1981). According to examination of the collected specimens, five species of *Schizothorax* belong to the Original group, two species of *Ptychobarbus* and one species of *Gymnodiptychus* belong to the Specialised group, and one species of *Gymnocypris* and one species of *Schizopygopsis* belong to the Highly specialised group. This result indicated that fishes of the Dingqu River Basin are crucial model species for evolutionary and conservation research of the Qinghai-Tibet Plateau.

Our sampling sites did not involve the upper Dingqu River due to its location at high altitude (> 4000 m) and difficult access by rugged mountain road. Upper and middle streams of the Mayihe River are located within the Tibetan Region and fish collection is not permitted in this area for religious reasons.

## Supplementary Material

11E009D9-84CF-5653-A584-CEC45CED413D 10.3897/BDJ.9.e76669.suppl1 Supplementary material 1 Collected fish taxon-occurrences of Dingqu River Data type taxon-occurrences Brief description This dataset provides taxonomic, distribution, habitat condition, endemism and threat information for each species collected from the Dingqu River Basin. File: oo_609325.tsv https://binary.pensoft.net/file/609325 authors of this paper

C704A928-8BA1-5161-B5B4-DB91ACEC8325 10.3897/BDJ.9.e76669.suppl2 Supplementary material 2 Historical fish taxon-occurrences of Dingqu River Data type taxon-occurrences Brief description This dataset provides taxonomic, distribution, habitat condition, endemism and threat information for each species collected from historical literature. File: oo_609326.tsv https://binary.pensoft.net/file/609326 authors of this paper
